# Cognitive Function in Urban and Rural Postmenopausal Women

**DOI:** 10.7759/cureus.58647

**Published:** 2024-04-20

**Authors:** Al Asmaul Husna, Munmun Mustafa, Ely Prue, Afsana Jahan, Nur-A-Safrina Rahman, Maisha Samiha, Tahsin Tasneem Tabassum, Syed E Shaude

**Affiliations:** 1 Department of Community Medicine, Pabna Medical College, Pabna, BGD; 2 Department of Community Medicine, Bangladesh Medical College, Dhaka, BGD; 3 Department of Community Medicine, Cox's Bazar Medical College, Cox's Bazar, BGD; 4 Department of Microbiology, Pabna Medical College, Pabna, BGD; 5 Department of Health, Projahnmo Research Foundation, Dhaka, BGD; 6 Department of Health and Nutrition, Safetynet Bangladesh, South Asia Field Epidemiology and Technology Network, Inc, Dhaka, BGD; 7 Department of Health Sciences, University of York, York, GBR; 8 Department of Research and Development, International Network of Doctors Journal, Dhaka, BGD

**Keywords:** women, rural, urban, cognitive function, postmenopausal, menopause

## Abstract

Background: Menopause is a well-known risk factor for decreasing cognitive function in women. Postmenopausal women are increasing in number but relevant studies are very scarce. This study compared the cognitive function between urban and rural postmenopausal women and assessed the influence of socio-demographic factors on cognitive function.

Objectives: The aim of the study was to assess the association between the cognitive function of urban and rural postmenopausal women.

Methods: This comparative cross-sectional study was conducted among 87 urban and 87 rural postmenopausal women who were selected by purposive sampling method from the Nakhalpara and Dhamrai area of Dhaka district during the period from January to December 2020. Data were collected with a semi-structured questionnaire based on the Bengali version of the Mini-Mental State Examination (MMSE) scale through face-to-face interviews and record reviewing with a checklist. Statistical analyses of the results were obtained using Microsoft Excel (Microsoft Corporation, Redmond, WA) and SPSS version 24 (IBM Corp., Armonk, NY).

Results: The mean age of postmenopausal women was 58.09 ± 8.163 years in urban areas and 60.00 ± 7.562 years in rural areas. The majority (31, 35.6%) of urban women were primary school pass whereas 58 (66.7%) rural women were illiterate. The mean family income of the women was 43022.99 ± 10992.57 Bangladeshi taka (BDT) in the urban group and 14022.99 ± 5023.14 BDT in the rural group. The study revealed that 31 (35.6%) women in the urban group and 53 (60.9%) women in the rural group had abnormal cognitive function.

Conclusion: The percentage of abnormal cognitive function was higher in rural postmenopausal women. Cognitive function has an association with monthly family income, housing condition, family type, age at the time of marriage, lifestyle, and co-morbidities. Policymakers can take the findings as a guide to formulate policies and programs for the improvement of cognitive function of postmenopausal women.

## Introduction

Cognitive function arises from the term cognition, which refers to the internal mental processes that include how people perceive, remember, speak, think, make decisions, and solve problems. Cognitive function describes many different functions such as memory and attention thought to be components of the mind [1]. Cognitive decline is a normal process of aging where there is a gradual decline in cognition beginning from early adulthood. Nearly all cognitive functions decline, on average, with age, but there is a large variability, which ranges from "successful" aging to dementia [2]. Elderly women have a higher risk of developing Alzheimer's disease (AD) than elderly men [3]. Menopause is a well-recognized risk factor that may accelerate cognitive aging in women. In middle-aged women, increasing age and menopause accelerated cognitive decline in processing speed, episodic memory, and working memory [4].

Women play an important role in replenishing the Earth but their reproductive capacity is not permanent; it ceases one day, which is coined as menopause. According to the World Health Organization (WHO), natural menopause is described as “permanent cessation of menstruation resulting from the loss of ovarian follicular activity,” and postmenopausal women who have experienced 12 consecutive months of amenorrhea. The worldwide natural menopausal age range is 45 to 55 years [5]. During this transition to menopause, women may experience many stressful events, which include both physical and psychological symptoms, including hot flashes, night sweats, irregular heartbeat, headaches, and sleep difficulties [6]. The prevalence of each of these symptoms related to menopause varies across ethnic and socioeconomic groups and between rural and urban women [7]. Menopausal women face discrimination because of their age, gender, and the fact that they live in rural areas.

Bangladesh is a developing country and due to improvements in the health status of the population, the life expectancy of women has become longer than men. Menopausal age is an important indicator of subsequent morbidity and mortality in women. Postmenopausal women have a significantly increased risk of cognitive impairment [8]. The prevalence of cognitive impairment increases with age in elderly populations [9]. Demographic factors and environmental factors may also be involved in cognitive impairment [10].

## Materials and methods

This cross-sectional study was carried out in the area of Nakhalpara and Ruail Union of Dhamrai Thana of Dhaka from January 2020 to December 2020. The study population was 174 rural and urban postmenopausal women. The study comprised two groups of urban and rural women, and a comparison was done between them. The inclusion criteria encompassed: (1) women who had experienced natural menopause; (2) women who had reached menopause in the last one year and above; (3) participants who gave informed written consent; and (4) women who resided within the selected area.

Postmenopausal women with acute confusional states, acute physical illnesses, and severe visual or hearing impairments were excluded from the study. Informed consent was taken from each patient before enrolment in this study. The respondents were interviewed face-to-face using a sociodemographic questionnaire. Cognitive impairment was assessed by the Bengali version of the Mini-Mental State Examination (MMSE). The MMSE [11] is a simple pen-and-paper test of cognitive function based on a total possible score of 30 points; it includes tests of orientation, concentration, attention, verbal memory, naming, and visuospatial skills. According to the MMSE, if the score is < 24, it indicates abnormal cognitive function, and if the score is ≥ 24, it indicates normal cognitive function on the basis of the single cutoff method.

The study was carried out in a tertiary healthcare facility and was approved by the National Institute of Preventive and Social Medicine (NIPSOM; Approval No.: NIPSOM/IRB/2020/1225). Statistical analyses of the results were obtained using Microsoft Excel (Microsoft Corporation, Redmond, WA) and SPSS version 24 (IBM Corp., Armonk, NY).

## Results

This cross-sectional study was conducted in the Department of Community Medicine, NIPSOM, Dhaka. The study population was 174 rural and urban postmenopausal women. The study comprised two groups of urban and rural women, and a comparison was done between them. After fulfilling the exclusion and inclusion criteria by purposive sampling method, a total of 174 apparent patients were included in the study. The data were expressed as mean ± SD, chi-square test, and t-test.

A total of 52 (59.8%) women in the urban group compared to 34 (39.1%) women in the rural group were in the age group of 45-59 years. About 22 (25.3%) women in the urban group compared to 39 (44.8%) women in the rural group were in the age group of 60-69 years (Table 1).

**Table 1:** Comparison of age between urban and rural postmenopausal women.

Age (years)	Postmenopausal women			Significance
	Urban, n (%)	Rural, n (%)	Total	
45-59	52 (59.8)	34 (39.1)	86 (49.4)	χ^2 ^= 8.542
60-69	22 (25.3)	39 (44.8)	61 (35.1)	
70-80	13 (14.9)	14 (16.1)	27 (15.5)	
Total	87 (100)	87 (100)	174 (100)	
Mean ± SD	58.09 ± 8.163	60.00 ± 7.562		t = -1.599

**Figure 1:**
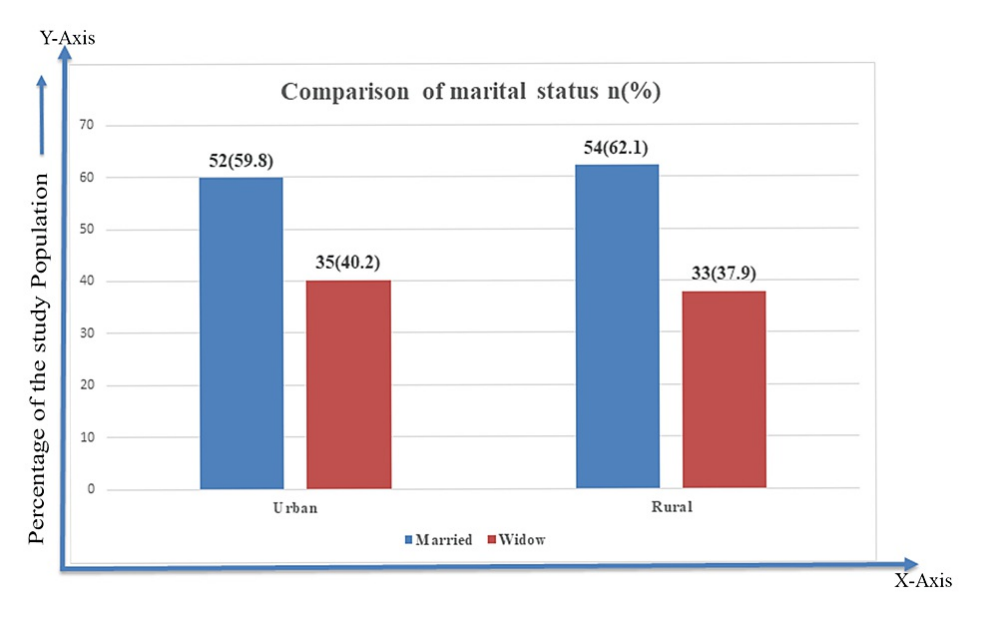
Comparison of marital status between urban and rural postmenopausal women.

Regarding marital status of postmenopausal women, the majority (54, 62.1%) in the rural group were married compared to 52 (59.8%) in the urban group. About 35 (40.2%) urban women were widows compared to 33 (37.9%) women in the rural group (Figure 1).

Regarding educational qualification, the majority (58, 66.7%) of postmenopausal women in the rural group were illiterate compared to 20 (23.0%) in the urban group. Also, only 31 (35.6%) postmenopausal women in the urban group studied up to the primary level compared to 26 (29.9%) women in the rural group. Regarding occupation, the majority (80, 92.0%) of postmenopausal women in the rural group were housewives compared to 68 (78.2%) postmenopausal women in the urban group. A total of 60 (69.0%) postmenopausal women in the urban group lived in pucca houses compared to seven (8.0%) women in the rural group. About 55 (63.2%) postmenopausal women lived in semi-pucca houses in the rural group compared to 27 (31.0%) in the urban group (Table 2).

**Table 2:** Comparison of educational qualification, occupation, and housing condition between urban and rural postmenopausal women.

	Postmenopausal women			Significance
	Urban, n (%)	Rural, n (%)	Total	
Educational qualification				
Illiterate	20 (23.0)	58 (66.7)	78 (44.8)	p = 0.000
Primary	31 (35.6)	26 (29.9)	57 (32.8)	
Secondary school certificate	28 (32.2)	3 (3.4)	31 (17.8)	
Higher secondary school certificate and above	8 (9.2)	0	8 (4.6)	
Occupation				
Housewife	68 (78.2)	80 (92.0)	149 (85.6)	p = 0.000
Business	6 (6.9)	0	6 (3.4)	
Service	13 (14.9)	0	13 (7.5)	
Day laborer	0	7 (8.0)	6 (3.4)	
Housing condition				
Bamboo, mud-built (kutcha)	0	25 (28.7)	25 (14.4)	p = 0.000
Walls of concrete and roof of hay or tin (semi-pucca)	27 (31.0)	55 (63.2)	82 (41.1)	
Building (pucca)	60 (69.0)	7 (8.0)	67 (38.5)	

Regarding the mean score of cognitive function of postmenopausal women, the mean ± SD of urban and rural postmenopausal women was 25.84 ± 4.430 and 23.43 ± 4.128, respectively (Table 3).

**Table 3:** Comparison of mean score of cognitive function between urban and rural postmenopausal women.

Attributes	Postmenopausal women		Significance
Mean score of cognitive function (range: 0-35)	Urban	Rural	
Mean ± SD	25.84 ± 4.430	23.43 ± 4.128	p = 0.000

A total of 56 (64.4%) postmenopausal women in the urban group had normal cognitive function compared to 34 (39.1%) in the rural group. About 31 (35.6%) and 53 (60.9%) of postmenopausal women had abnormal cognitive function in urban and rural groups, respectively. A total of 82 (94.3%) postmenopausal women in the rural group were aged 10-17 years at the time of marriage while 61 (70.1%) women in the urban group fell into that group. A total of 26 (29.9%) postmenopausal women in the urban group in comparison to five (5.7%) postmenopausal women in the rural group were aged 18-30 years at the time of marriage. A total of 75 (86.2%) postmenopausal women in the urban group were aged 10-12 years at the time of menarche while 67 (77.0%) postmenopausal women in the rural group fell into that group. A total of 12 (13.8%) women in the urban group and 20 (23.0%) in the rural group fell into the 13-15 years age group at the time of menarche. A total of 81 (93.1%) women in the urban group fell into the group of 45-50 years while 79 (90.8%) women in the rural group fell into that group. A total of six (6.9%) and eight (9.2%) postmenopausal women fell into the 51-55 years age group in urban and rural groups, respectively (Table 4).

**Table 4:** Comparison of level of cognitive function, age at the time of marriage, age of menarche, and age at the onset of menopause between urban and rural postmenopausal women.

	Postmenopausal women			Significance
	Urban, n (%)	Rural, n (%)	Total	
Level of cognitive function				
Normal (≥24)	56 (64.4)	34 (39.1)	90 (51.7)	p = 0.001
Abnormal (<24)	31 (35.6)	53 (60.9)	84 (48.3)	
Age at the time of marriage				
10-17	61 (70.1)	82 (94.3)	143 (82.2)	p = 0.000
18-30	26 (29.9)	5 (5.7)	31 (17.8)	
Total	87 (100)	87 (100)	174 (100)	
Mean ± SD	16.21 ± 3.625	13.54 ± 2.219		
Age of menarche				
10-12	75 (86.2)	67 (77.0)	142 (81.6)	0.117
13-15	12 (13.8)	20 (23.0)	32 (18.4)	
Total	87 (100)	87 (100)	174 (100)	
Mean ± SD	11.61 ± 0.992	12.13 ± 1.274		0.003
Age at the onset of menopause				
45-50	81 (93.1)	79 (90.8)	160 (92.0)	p = 0.577
51-55	6 (6.9)	8 (9.2)	14 (8.0)	
Total	87 (100)	87 (100)	174 (100)	
Mean ± SD	48.80 ± 2.011	48.06 ± 2.115		0.018

Sixteen (18.4%) women in the urban group had diabetes mellitus compared to 12 (13.8%) in the rural group. Among postmenopausal women, one (1.1%) in the urban group had a stroke compared to two (2.3%) in the rural group. Among postmenopausal women, 53 (60.9%) in the urban group had hypertension compared to 50 (57.5%) in the rural group. A total of 67 (77.0%) women in the rural group had substance abuse to chewing betel nuts compared to 42 (48.3%) women in the urban group (Table 5).

**Table 5:** Comparison of co-morbidities and substance abuse between urban and rural postmenopausal women.

	Postmenopausal women				Significance
	Urban, n (%)		Rural, n (%)		
	Yes	No	Yes	No	
Co-morbidities					
Diabetes mellitus	16 (18.4)	71 (81.6)	12 (13.8)	75 (86.2)	p = 0.409
Stroke	1 (1.1)	86 (98.9)	2 (2.3)	85 (97.7)	p = 1.000
Hypertension	53 (60.9)	34 (39.1)	50 (57.5)	37 (42.5)	p = 0.644
Substance abuse					
Betel nuts	42 (48.3)	45 (51.7)	67 (77.0)	20 (23.0)	p = 0.000
Zorda (as zorda is ready to use tobacco, it induces intoxication)	37 (42.5)	50 (57.5)	36 (41.4)	51 (58.6)	p = 0.878
Sadapata (as sadapata is ready to use tobacco, it induces intoxication)	2 (2.3)	85 (97.7)	26 (29.9%)	61 (70.1)	p = 0.000

A total of 51 (91.1%) women had a normal condition in the 45-59 years group and one (3.2%) had an abnormal condition in that age group. In the 60-69 years age group, five (8.9%) had a normal condition and 17 (54.8%) had an abnormal condition. Within the rural postmenopausal women, the maximum (33, 62.3%) had an abnormal condition in the 60-69 years group and six (17.6%) had a normal condition in that age group. A total of 49 (87.5%) married women had a normal cognitive function and three (9.7%) married women had an abnormal cognitive function. In the case of widows, seven (12.5%) had a normal cognitive function and 28 (90.3%) had an abnormal cognitive function. In comparison to rural postmenopausal women, the maximum (27, 50.1%) married women had an abnormal cognitive function and 27 (79.4%) married women had a normal cognitive function. Seven (12.5%) had a normal cognitive function and 13 (41.9%) had an abnormal cognitive function in the illiterate group. In the case of the primary-level education group, 14 (25.0%) had a normal cognitive function and 17 (54.8%) had an abnormal cognitive function. In comparison to rural postmenopausal women, the majority (15, 44.1%) had a normal cognitive function and 43 (81.1%) had an abnormal cognitive function in the illiterate group. A total of 53 (94.6%) women had a normal function in the 1-10 years of menopause group and four (12.9%) had an abnormal function in that group. In the 11-20 years of menopause group, three (5.4%) had normal and 18 (58.1%) had abnormal cognitive function. In comparison to the rural postmenopausal women, the maximum (32, 60.4%) had an abnormal condition in the 11-20 years of menopause group and 27 (79.4%) had a normal condition in that group. A total of 52 (92.9%) women who took food on time had a normal cognitive function and 25 (80.6%) had an abnormal function. Also, of those who did not take food on time, four (7.1%) had normal and six (19.4%) had abnormal cognitive function. In comparison to the rural postmenopausal women, the maximum (30, 88.2) had a normal condition and 33 (62.3%) had an abnormal condition. A total of 40 (71.4%) women who did not chew betel nuts had a normal cognitive function and 26 (83.9%) who chew betel nuts had abnormal function. In comparison to the rural postmenopausal women, the maximum (48, 90.6%) women who chew betel nuts had abnormal cognitive (Table 6).

**Table 6:** Comparison of the level of cognitive function by different variables between urban and rural postmenopausal women.

Types of postmenopausal women	Level of cognitive function (urban)			Level of cognitive function (rural)		
	Normal (≥24)	Abnormal (<24)	Significance	Normal (≥24)	Abnormal (<24)	Significance
Age group (years)						
45-59	51 (91.1)	1 (3.2)	p = 0.000	27 (79.4)	27 (50.1)	p = 0.004
60-69	5 (8.9)	17 (54.8)		7 (20.6)	26 (49.9)	
70-80	0	13 (41.9)		34 (100)	53 (100)	
Marital status						
Married	49 (87.5)	3 (9.7)	p = 0.000	7 (20.6)	26 (49.9)	p = 0.008
Widow	7 (12.5)	28 (90.3)		34 (100)	53 (100)	
Educational qualification						
Illiterate	7 (12.5)	13 (41.9)	p = 0.000	15 (44.1)	43 (81.1)	p = 0.001
Primary	14 (25.0)	17 (54.8)		17 (50.0)	9 (17.0)	
Secondary school certificate	27 (48.2)	1 (3.2)		2 (5.9)	1 (1.9)	
Higher secondary school certificate and above	8 (14.3)	0		0	0	
Duration of menopause (years)						
1-10	53 (94.6)	4 (12.9)	p = 0.000	27 (79.4)	12 (22.6)	p = 0.000
11-20	3 (5.4)	18 (58.1)		7 (20.6)	32 (60.4)	
21-30	0	9 (29.0)		0	9 (17.0)	
Take food in time						
Yes	52 (92.9)	25 (80.6)	p = 0.087	30 (88.2)	33 (62.3)	p = 0.007
No	4 (7.1)	6 (19.4)		-	-	
Substance abuse to chewing betel nuts						
Yes	16 (28.6)	26 (83.9)	p = 0.000	19 (55.9)	48 (90.6)	p = 0.000
No	40 (71.4)	5 (16.1)		15 (44.1)	5 (9.4)	

## Discussion

Socio-demographic characteristics between urban and rural postmenopausal women

This study is the first to demonstrate a significant rural-urban difference in cognitive function of postmenopausal women in Bangladesh. The mean age for the urban group was 58.09 ± 8.163 years and the mean age for the rural group was 60.00 ± 7.562 years. The value explained that urban and rural groups were almost properly matched on the basis of age. The study found that the majority (59.8%) of postmenopausal women in the urban group and 39.1% in the rural group were aged 45-59 years. Age has a significant value for cognitive function. A study in Japan reported quite dissimilar findings where the urban group had 32.4% and the rural group had 36.4% of women aged ≥65 years [11]. This is due to the sample size and study population dissimilarity between the two studies.

Nearly half (44.8%) of the study subjects were literate. Significantly, illiteracy was higher among the rural postmenopausal women (66.7%) as compared to the urban elderly (23.0%). The majority (85.6%) of women were housewives. Significantly, housewives were more among the rural postmenopausal women (92.0%) as compared to the urban elderly (78.2%). Most (47.1%) of them lived in semi-pucca houses. Most (55.2%) of them had family members ranging from four to six members.

The majority (82.2%) of women were aged 10-17 years at the time of marriage. Significantly, the age of 10-17 years at the time of marriage was more among the rural postmenopausal women (94.3%) as compared to urban elderly (70.1%). The majority (81.6%) of women's age of menarche was 10-12 years. The majority (92.0%) of women's age at the onset of menopause was 45-50 years. Most (55.2%) of them had a duration of menopause of one to 10 years. The majority (60.9%) of women suffered from hypertension (HTN) and 18.4% suffered from diabetes mellitus (DM). Another study in Bangladesh found that 95.3% suffered from hot flashes and 77.3% chewed betel nuts [12]. This is quite similar due to geographical similarity.

Cognitive function between urban and rural postmenopausal women

In this study, regarding age, the majority (54.8%) had an abnormal cognitive function in the 60-69 years group, and 91.1% had a normal cognitive function in the 45-59 years group in the urban women. The majority (82.4%) had a normal cognitive function in the 45-59 years group and 62.3% had an abnormal cognitive function in the 60-69 years group in rural women. A study showed that increasing age increased the risk of cognitive impairment. The study was to reify that age changes within individuals over time (and age) can be inferred from cross-sectional age differences between groups of individuals of different agents measured at the same time point [13]. This is quite similar due to socio-economical similarity. A study by Sharma et al. found that marital status (odds ratio = 7.8) significantly predicts cognitive impairment in the elderly and said that widowed elderly have more severe cognitive impairment as compared to married elderly. This is quite similar due to socio-economical similarity [14].

In the case of education, the majority (81.1%) of illiterate women who had abnormal cognitive function belonged to the rural group. Another study showed that participants with high school or higher (8.2%) and primary education had one-fifth and one-fourth likelihood of cognitive impairment than illiterates (39.6%) [15]. This is due to socio-economical similarity.

Cognitive function with other attributes between urban and rural areas

Regarding co-morbidities, logistic regression showed that HTN and DM increased the risk of cognitive impairment, with coefficient = 2.292, 95% CI = 4.761-20.583, and p = 0.000 for HTN and coefficient = 1.858, 95% CI = 2.308-17.803, and p = 0.000 for DM. A study reported that cognitive impairment was associated with an increase in morbidity [16]. This similarity may be due to similarity in age. A study done by Goodwin reported that nutritional risk is associated with cognitive impairment in the elderly [17]. This is quite similar due to the similarity in socioeconomic conditions.

The limitation of the study was the sample was collected purposively, so bias might occur, and the sample size was small.

## Conclusions

Cognitive function impairment is a common health problem in postmenopausal women. However, limited information regarding this topic is found. For this reason, the study was done to determine the association of cognitive function between urban and rural postmenopausal women. Regarding cognitive function among rural postmenopausal women, more than half had an abnormal cognitive function but among urban postmenopausal women, more than one-fourth had an abnormal cognitive function. Abnormal cognitive function was higher in the rural group, which was statistically significant.
